# Clinical and Therapeutical Importance of HBV genotyping in Romania 

**Published:** 2008-04-15

**Authors:** I Constantinescu, F Nedelcu, MA Toader, D Vasile

**Affiliations:** Centre for Immunogenetics and Virology Fundeni Clinical InstituteRomania

## Abstract

In a country with a high prevalence (16%) of chronic serum HBsAg carriers like Romania there is a special interest in the diagnosis, epidemiology, clinics, pathology and treatment of HBV infection

The idea of HBV genotyping arose from the need of understanding the complex interactions between virus and host.

The purpose of this article is to present a study which aimed to identify the circulating HBV genotypes in Romania, correlate them with the clinical outcome and by HBV genotyping, to make a selection of patients for the most appropriate antiviral therapy.

130 patients were selected, from different areas of the spectrum of HBV infection in which a quantitative determination of HBV–DNA was performed.

HBV A genotype is associated with the inactive carrier status; a symptomatic HBV–HDV was identified in the double infection. The HBV D genotype has the most common HBV genotype (66%) and is associated with active viral infection and hepatocellular carcinoma. Long term HBV chronic infection revealed a mixture of A and D genotypes in most cases.

For a proper selection of patient for the antiviral therapy, we should mandatorily genotype the HBV virus before the onset of treatment and all genotyping data must be correlated with liver biopsy assessments.

Keywords: HBV genotyping, core promoter, precore variant, antiviral therapy

The worldwide health problem of chronic liver diseases caused by persistent infection with hepatitis B virus (HBV) has determined the rapid development of therapeutical strategies created in order to control the process of active viral replication and prevent subsequent clinical consequences such as cirrhosis and hepatocellular carcinoma.

Chronic HBV hepatitis centers the attention of modern hepatology as there are an impressive number of 400 x 106 chronic carriers, HBV infection being the leading cause of liver cirrhosis and hepatocellular carcinoma [1]. It is estimated that approximately 1.1 million people die every year from HBV infection [2, 10].

The initial stages of hepatitis virus research in Romania can be easily defined and timelined, but later stages are harder to outline, since they overlap. Combined and complex methodologies involving more and more fields – virology, hepatology and immunology, have lead to the valid results that represent the basis of the huge edifice of current knowledge on viral hepatitis.

In Romanian virology, study of viral hepatitis is an old tradition; for Stefan S. Nicolau and Nicolae Cajal researches on viral hepatitis represented a preferential subject, managing to formulate numerous ethiopathogenic concepts with high–priority worldwide diagnostics and introduce the concept of pluriethiology of viral hepatitis [3].

Viral hepatitis has been and continues to be an important issue in Romanian medical literature especially in respect to clinical aspects.

Viral hepatitis represents a major public health problem in our country and constitutes for virologists the most dynamic objective of study and contemporary research.

In a country with a high prevalence (16%) of chronic serum HBsAg carriers like Romania there is a special interest in the epidemiology, clinics, pathology and treatment of HBV infection [4].

HBV carries out the replication of its DNA genome through a process of reverse transcription with shortcomings in the reading process, resulting in the HBV quasispecies.

HBV A and D genotypes have different replication efficiencies in cell cultures and these characteristics are kept in vivo [9]. Often, the mutant variants, especially those in the ‘precore’ and ‘core promoter’ regions predominate together with the wild–type HBV in the same host.

For the viral dominance, both intra–viral population and intra–host factors must be taken into account. Mutants can develop a selective advantage within the infected host during chronic infection. Negative HBeAg mutants have a biological advantage over the wild–type viral strains, so these strains are selected through the process of evolution

The idea of HBV genotyping arose from the need of understanding the complex interactions between virus and host.

In the 80's, Vincent Babeş carried out the first study on HBV serotyping in Romania, showing that the prevalent subtypes are: adw and ayw [3].

At that time, Babes sustained that the clinical and prognostic value of HBV subtypes was controversial, stressing the necessity for guided research based on a unitary and standardized methodology.

HBV is a noncytopathic virus, being the prototype virus of the Hepadnaviridae family. HBV is an enveloped virus with a circular DNA genome, partially double–stranded, of about 3.2 kbp. (Fig 1)

**Fig 1:**
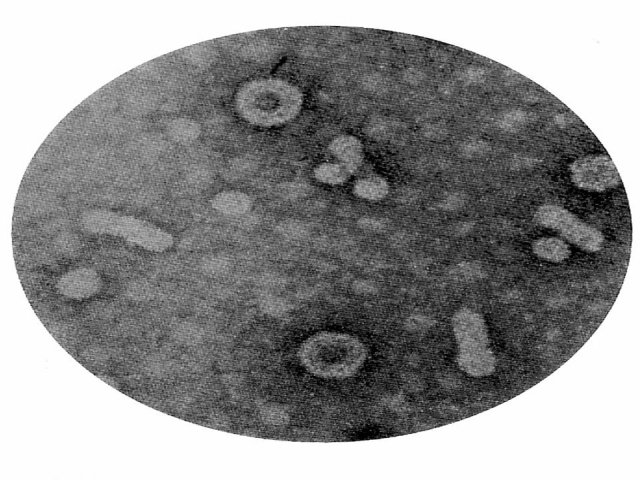
HBV – The Hepadnaviridae family prototype

Thus, after hepatocyte infection, genomic HBV DNA is transported into the nucleus, where it is transformed into a covalently closed circular DNA molecule (cccDNA) [14]. cccDNA determines the transcription of viral mRNA for the generation of viral gene products. One of the viral mRNA molecules is larger than the genome and encodes for the capsid proteins [14].

After its translocation, the ‘core’ protein will encase its own mRNA, known as pregenomic RNA to form the capsid [4].

The pregenomic RNA is subsequently converted to the partially double–stranded DNA genome by the DNA polymerase. The ‘core’ particle will interact with the proteins of the viral envelope that had been inserted into the membrane of the endoplasmic reticulum (ER). This interaction allows the ‘core’particle to enter the lumen of the ER and obtain its own envelope [4]. This viral particle released into the lumen of the ER is then secreted from the host cell. During the viral replication cycle three major viral antigens are produced, the ‘core’ antigen, the ‘surface’ antigen and the ‘e’ antigen [14]. The HBV genome contains three open reading frames (ORF) that have overlapping regions and encode the ‘core’ proteins, surface antigens, DNA polymerase and a transcriptional transactivator known as protein X [4].

The pre–S/S region has three starting points so that three forms of HBsAg are encoded: the proteins of the large envelope (L), of the medium envelope (M) and the small envelope (S). 

The ‘precore–core’ region has two starting codons that produce two different proteins: HBeAg and HBcAg, the structural component of the nucleocapsid.

The polymerase gene encodes a multifunctional enzyme with multifunctional enzymatic activities of reverse transcriptase, DNA polymerase and RNase.

The X protein is a transactivator and can play a role in carcinogenesis.

HBV replication allows reading errors of the ORF, thus resulting in variations of the initial infectant HBV strain. During the infection, variations of HBV sequences can be seen in almost all of the regions of the HBV genome. [15]. As a result of this phenomenon, HBV circulates in the form of quasispecies in infected patients, who carry different strains and genotypes [9].

Currently, 8 different HBV genotypes have been identified with a distinct geographic distribution. Thus, genotype A (adw serotype) and D (ayw serotype) are frequently found in the United States and Europe [12]; genotype B (adw) and C (adr) are prevalent in China and South–East Asia [6]. In Romania, the prevalent serotypes were identified in the 80's by Vincent Babes and were adw and ayw [3].

Recent data suggests that there are differences in the clinical evolution and in the sustained virological response to antiviral therapy which is closely correlated with the HBV genotype.

Thus, the A HBV genotype was associated with a favorable disease evolution and long–term survival [11]. The B HBV genotype was associated with the development of hepatocellular carcinoma. C HBV genotype was associated with severe disease progression [6, 11].

Keeping in mind these aspects, we were interested in genotyping HBV, continuing the work of our virology professors and gaining a better understanding of the virus–host relationship, the sustained response to antiviral therapy, the outcome of chronic infection and the progression to hepatocellular carcinoma.

## Goals of the study

- to identify the circulating HBV genotypes in Romania
- to correlate HBV genotypes with clinical outcome
- by HBV genotyping, to make a selection of patients for the most appropriate antiviral therapy

## Patients

A number of 130 patients were selected, divided in the following groups: 35 serum HBsAg asymptomatic carriers, with serum HBeAb positive, nonsignificant viral replication, normal ALT and minimal liver tissue changes; 15 patients with chronic hepatitis, serum HBsAg and HBeAb, low to moderate viral replication, high serum level of ALT, HDV coinfection and aggressive liver histology features (Figure 3); 68 patients with serum HBsAg and HbeAb positive, high serum levels of ALT, and high viral replication having aggressive liver biopsy characteristics; 12 patients with serum HBsAg and HBeAb positive, associated with hepatocellular carcinoma, high serum levels of ALT, high viral replication, high serum levels of alpha–fetoprotein (AFP) and hepatocellular carcinoma changes in liver.

## Materials and Methods

All patients were tested for the presence of HBsAg, HBcAb, HBsAb, HBeAg and HBeAb in the serum. The quantitative determination of HBV–DNA was performed with HBV 2.0 Monitor (Roche Molecular Diagnostics).

HBV genotyping was performed using the INNO–LiPA DR amplification kit and INNO–LiPA genotyping kit (Innogenetics).

The amplification reaction preceding the genotyping process is a PCR reaction, using ‘outer’ and ‘nested’ biotinylated oligonucleotide primers, consequently generating biotinylated amplified DNA of the HBV–pol gene of strains B to C and obtaining 10 micro L of HBV, DR amplification product in the open reading frame of the polymerase gene at codon 180 – 204 – 207.

INNO–LiPA Test principle: the test is based on the reverse hybridization principle: amplified biotinylated DNA material is chemically denatured, and the separated strands are hybridized with specific oligonucleotide probes immobilized as parallel lines on membrane – based strips. This is followed by a stringent wash step to remove any mismatched amplified material. After the stringent wash, streptavidin conjugated with alkaline phosphatase is added and bound to any biotinylated hybrid previously formed. Incubation with a substrate solution containing a chromogen results in a purple/brown precipitate.

The reaction is stopped by a wash step, and reactivity pattern of the probes is recorded.

## Results

**Fig 2:**
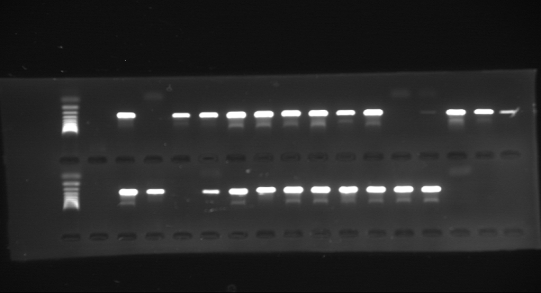
HBV–DNA amplicons

The HBV – DNA amplicons are shown in Fig 2

The HBV genotypes identified for the 130 selected patients were:

- **A HBV Genotype – 6 patients representing 5%**
- **D HBV Genotype – 15 patients representing 12%**
- **A HBV and D HBV Genotype– 97 patients representing 75%**
- **(A+D+F; D+F) HBV Genotype combinations –12 patients representing 9%**

Fig 3, Fig 4

**Fig 3:**
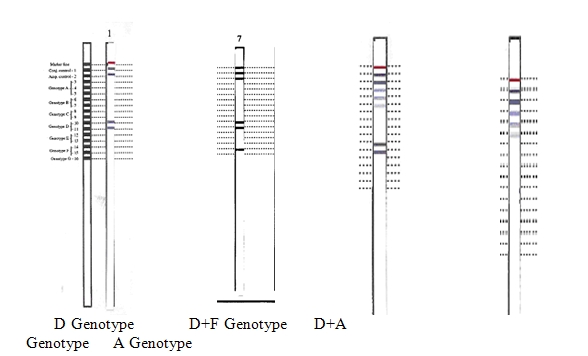
HBV genotyping results

**Fig 4:**
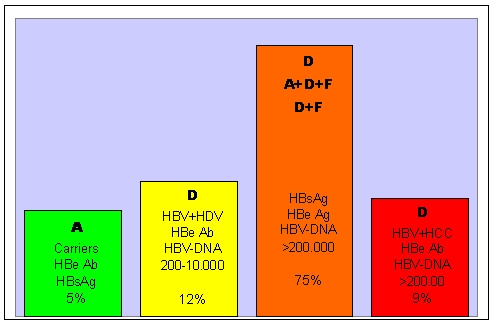
HBV genotypes in 130 chronic HBV patients

These data correlate with the clinical, biochemical and histopathological status of patients.

**Fig 5:**
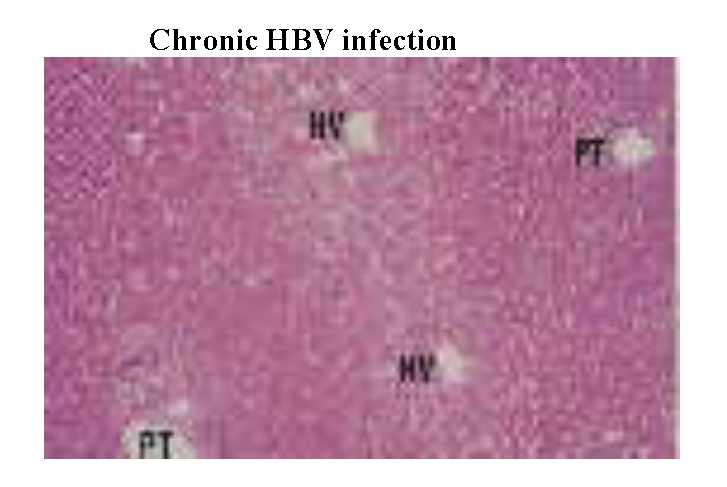
Section of liver showing normal architecture with regularly spaced portal tract (PT) and hepatic venues (HV). Haematoxylin and eosin stain; magnification approximately x35. (16)

**Fig 6:**
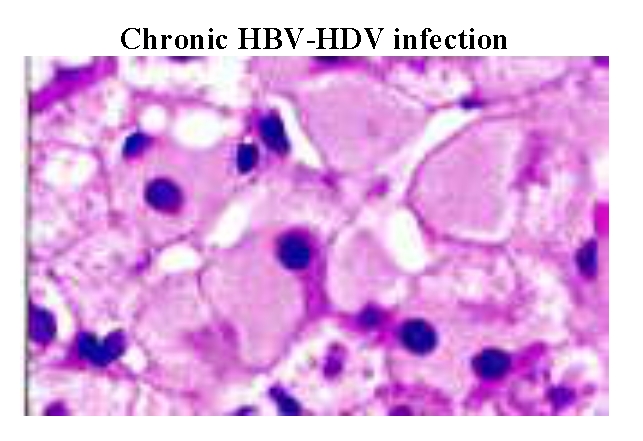
Low HBV – DNA replication associated with ‘ground–glass’ hepatocytes. Cells with a large amount of HBsAg in the cytoplasm appear pale and uniformly pink with haematoxylin and trs/sin stain. (16)

**Fig 7:**
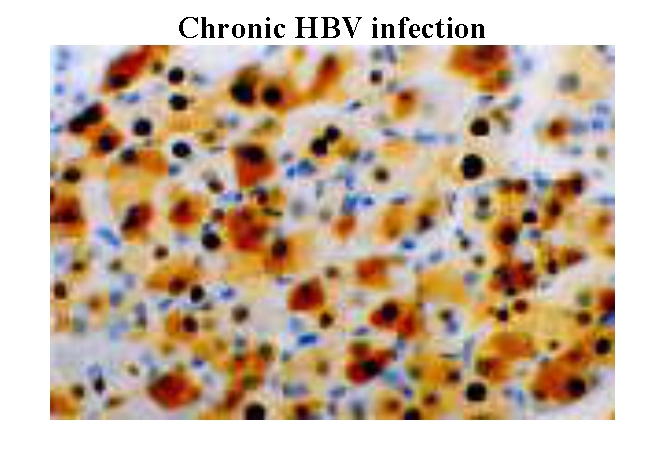
High viral replication associated with the presence of HBcAg (core antigen) in nuclei and cytoplasm (brown staining) (16)

**Figure 8:**
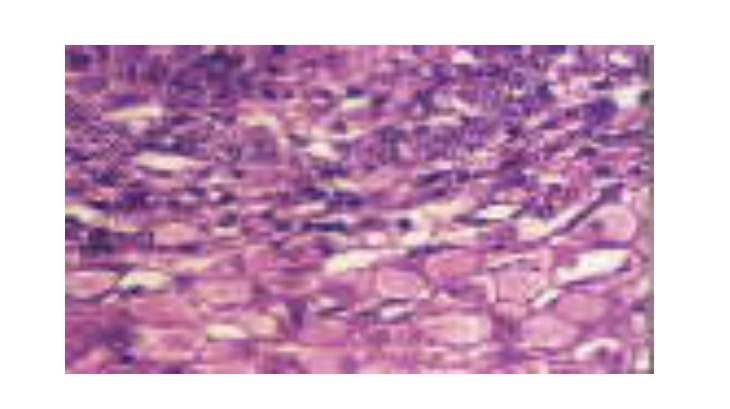
HBV–associated HBsAg hepatocellular carcinoma. Hepatocellular carcinoma cells distinguished by an increased nucleocytoplasmic ratio and a darker staining pattern. Numerous cells have a ‘ground glass’ appearance, resulting from the expression of HBsAg. Magnification approximately x150. (16)

The conclusions of the HBV genotyping study are: HBV A genotype is associated with the inactive carrier status; a symptomatic HBV–HDV was identified in the double infection. The HBV D genotype has the most common HBV genotype (66%) and is associated with active viral infection and hepatocellular carcinoma. (Fig 5, Fig 6, Fig 7, Fig 8)

Long term HBV chronic infection revealed a mixture of A and D genotypes in most cases.

## Discussion

Currently, mutations are known to occur during chronic HBV infection at the nucleotide sequence level of HBV genome and have important clinical and virological case consequences.

Thus, a mutation in the S gene that includes the substitution of glycine with arginine at codon 145 (G145R) was frequently found in new–born from HBsAg–positive mothers, for whom the HBV infection evolved in spite of vaccination at birth. In addition, this mutation was detected in patients with liver transplant that developed recurrent HBV infection, despite the therapy with HIBg immunoglobulin. The ‘core promoter’ and ‘precore’ variants produce HBV virions that do not secrete HBeAg [5].

The most frequent ‘precore promoter’ mutation has a dual alteration A1762T and G1764A that diminishes ‘precore’ mRNA and thus the secretion of HBeAg [8, 15].

These variants are found particularly in patients that do not have serum HBeAg, belonging to B, C and D genotypes. The G1896A premature stop codon mutation should also be mentioned [13].

In severe acute and chronic hepatitis cases ‘precore’ HBV variants have been described [5].

Mutations in the polymerase gene were identified in patients undergoing antiviral therapy who develop antiviral resistance. The best characterized mutants in the polymerase gene were identified during lamivudine therapy.

M552V, M552I, L528M, (YMDD) mutations occur in the catalytic domain of the polymerase providing resistance to lamivudine and related nucleosidic analogs [7].

Preliminary sequencing data of circulating HBV strains in Romania were determined by our working group together with our colleagues from London, UK (T.J. Harrison, R. Ling, I. Constantinescu – unpublished data, 2000).

The patients with chronic HBV infection that were selected for sequencing had different clinical outcomes of the disease, either aggressive or biochemically and histologically mild. Our target was to find a possible correlation between eventual HBV ‘core promoter’ mutants and clinical and paraclinical aspects.

Preliminary data showed in the high replicative and aggressive chronic HBV infection cases mutations of the stop codon type: 28 Tryptophan–> stop codon; 131 Arginine–> Lysine (aac –> aaa); 145 Glycine –> Arginine (gga–> aga).

This study is in progress and the final results will offer new aspects of ‘core promoter’ mutations and the clinical and histopathological evolution of HBV–induced chronic infection.

The genotyping data presented above showed a closely correlation between HBV genotypes and clinical and histopatological outcome.

Genotype D HBV is associated with long term survival and the chronic carrier status of HBV infection. 

Genotype D expresses a high replicative chronic HBV infection with serious histological changes.

Genotype D–seems to be in associated with the development of HCC.

Long term chronic HBV infection is characterized by a mixture of (A+D+F) HBV genotypes.

For a proper selection of patient for the antiviral therapy, we should mandatorily genotype the HBV virus before the onset of treatment.

All genotyping data correlated with liver biopsy assessments. ( Fig 5, Fig 6, Fig 7, Fig 8)

For A HBV genotyping the most suitable attitude should by follow–up.

For genotype D the most adequate treatment should be nucleoside analogs (lamivudine, adefovir, entecavir).

For the mixed A+D HBV genotype, combination therapy–nucleoside analogs and interferon should be the best therapeutical attitude.

In the general population wild–type HBV still predominates over other mutant forms.

Furthermore, the stop codon mutant form in the ‘precore’ region is associated with fulminant hepatitis and is much more frequently encountered in the D genotype of HBV [5]. In Romania, the prevalent genotype of HBV is D and it is important to genotype and find mutations in the viral genome in order to choose the most adequate antiviral therapy and provide the most accurate predicted outcome.

In such cases, the therapy of choice is lamivudine, which acts on the dominant ‘precore’ mutant variant.

It is known that IFN has a potential antiviral activity only on the wild type strain. Therefore, the therapeutic decision must be taken on a case–by–case basis, only after a molecular virology diagnostic, in order to obtain a sustained virological answer, in fact, a therapeutic success.

## Conclusions 

HBV molecular virology studies provide a unique understanding of the virus–host relationship with an important impact on the evolution of the hepatitis infection and its possible outcome, but especially on the selection of patients for antiviral therapy.

In conclusion, without a molecular virology diagnosis we cannot successfully understand and treat a chronic HBV infection.
